# Healthcare access barriers for FARC ex-combatants in Colombia: qualitative perspectives from healthcare providers and FARC health promoters

**DOI:** 10.1186/s12889-020-10062-3

**Published:** 2021-01-08

**Authors:** Christopher W. Reynolds, Leonar G. Aguiar, Christian Arbelaez, Carlos Gómez Restrepo, Andres Patiño, Heidy Carranza, Lindsey Pileika, Andrés Duarte

**Affiliations:** 1 University of Michigan, 1500 E Medical Center Dr, Ann Arbor, MI 48109 USA; 2 Pontificia Universidad Javeriana, Hospital Universitario San Ignacio, Cra. 7 #No. 40 -62, Bogotá, Colombia; 3 Brown Emergency Medicine, 125 Whipple St # 3rd, Providence, RI 02908 USA; 4 Emory University School of Medicine, 550 Peachtree St. NE, Atlanta, GA 30308 USA; 5 Warren Alpert Medical School of Brown University, 222 Richmond St, Providence, RI 02903 USA

**Keywords:** Healthcare access barriers, FARC ex-combatant, Colombia, Global health, Armed conflict, Reintegration

## Abstract

**Background:**

Following the 2016 Peace Agreement with the *Fuerzas Armadas Revolucionarias de Colombia* (FARC), Colombia promised to reincorporate more than 13,000 guerrilla fighters into its healthcare system. Despite a subsidized healthcare insurance program and the establishment of 24 *Espacios Territoriales de Capacitación y Reincorporación* (ETCRs—Territorial Spaces for Training and Reintegration) to facilitate this transition, data has shown that FARC ex-combatants access care at disproportionately lower rates, and face barriers to healthcare services.

**Methods:**

Semi-structured interviews were conducted with FARC health promoters and healthcare providers working in ETCRs to determine healthcare access barriers for FARC ex-combatants. Analysis was completed with a qualitative team-based coding method and barriers were categorized according to Julio Frenk’s Domains of Healthcare Access framework.

**Results:**

Among 32 participants, 25 were healthcare providers and 7 self-identified as FARC health promoters. The sample was majority female (71.9%) and worked with the FARC for an average of 12 months in hospital, health center, medical brigade, and ETCR settings. Our sample had experiences with FARC across 16 ETCRs in 13 Departments of Colombia. Participants identified a total of 141 healthcare access barriers affecting FARC ex-combatants, which affected healthcare needs, desires, seeking, initiation and continuation. Significant barriers were related to a lack of resources in rural areas, limited knowledge of the Colombian health system, the health insurance program, perceived stigma, and transition process from the FARC health system.

**Conclusions:**

FARC ex-combatants face significant healthcare access barriers, some of which are unique from other low-resource populations in Colombia. Potential solutions to these barriers included health insurance provider partnerships with health centers close to ETCRs, and training and contracting FARC health promoters to be primary healthcare providers in ETCRs. Future studies are needed to quantify the healthcare barriers affecting FARC ex-combatants, in order to implement targeted interventions to improve healthcare access.

## Background

Colombia is a country that has faced challenges in achieving health equity, particularly in rural areas due to longstanding and complex internal conflicts [1]. Following the 2016 Peace Agreement between the government and *Fuerzas Armadas Revolucionarias de Colombia* (FARC), Colombia made significant strides towards ending the longest war in the Western Hemisphere by promising to reincorporate more than 13,000 guerrilla fighters [2]. The United Nations has prioritized the healthcare status of ex-combatants as a key aspect of their guidelines for Disarmament, Demobilization, and Reintegration [3], and the importance of healthcare access in reintegration has been highlighted by similar examples in Nicaragua [4], Liberia [5], Rwanda [6], Sierra Leone, Afghanistan, and Ivory Coast [7]. As such, a cornerstone aspect of FARC reincorporation was access to healthcare, offered by a specialized health insurance provider, or EPS (*Entidad Promotora de Salud)* at little cost to ex-combatants [8]. The FARC (or Farian) community was estimated to have disproportionately high healthcare needs due to unmanaged chronic conditions, infectious diseases, and undiagnosed mental health trauma from the conflict [9–11]. The peace agreement with the FARC promised to strengthen health systems in rural areas where healthcare access has historically been limited [12]; including reinforcing infrastructure, implementation of telemedicine, differential approaches for gender-specialized care, and a focus on prevention and health promotion [8]. With this agreement, 24 *Espacios Territoriales de Capacitación y Reincorporación* (ETCRs), or Territorial Spaces for Training and Reintegration, were established in 16 Departments of Colombia [9], where approximately 8200 FARC have lived and utilized reincorporation services offered by the Colombian government [13].

Prior studies have shown that FARC are not accessing the healthcare system at proportionate rates and face significant barriers to care. EPS data based on FARC healthcare appointments showed that while 10,363 (74%) were registered to the health insurance system, less than one-third (3235) of affiliated FARC had accessed the healthcare system in the first year [10]. In comparison with the general population, a study showed that 63% of Colombians access institutionalized healthcare per year solely for prevention measures [14], indicating that FARC ex-combatants are accessing healthcare at markedly lower rates. A 2017 report from *Defensoria del Pueblo de Colombia* showed insufficient levels of healthcare resources, personnel, and services across ETCRs, including certain areas with no reported healthcare resources or institutions at all [15]. In 2019, a quantitative survey in ETCRs showed 75% of FARC were able to access a health center or necessary medications and highlighted high rates of emergency and obstetric care needs [16]. Finally, there are anecdotal examples of preventable deaths among the Farian community due to healthcare access difficulties [17], and isolated reports of FARC being denied healthcare by providers and institutions [18]. These medical, social and cultural barriers prevent FARC ex-combatants from achieving equitable healthcare access, contribute to preventable morbidity and mortality among these communities, and threaten peace building in these vulnerable regions of Colombia.

It is known that there are gaps in healthcare access for FARC ex-combatants. However, few studies have defined and categorized the specific barriers for this community, and very little exists in the form of qualitative studies to describe them. This study aimed to understand the perspective of FARC health promoters and certified healthcare providers working in the ETCRs across Colombia, to identify and categorize barriers to accessing healthcare services for FARC ex-combatants.

## Methods

### Study design

A semi-structured, preliminary interview script based in exploratory methodology was developed from a literature review of qualitative studies on healthcare access barriers for vulnerable populations. The script was pilot tested with focus groups composed of FARC community health promoters and physicians who had cared for FARC communities (*n* = 5) to generate themes and purge superfluous questions. The final script lasted approximately 45 min, and consisted of six sections including demographics, perceived healthcare barriers, FARC medical difficulties, and suggestions for addressing barriers (Additional file 1, Spanish; Additional file 2, English).

### Data collection and population

During a 3-month period in 2019, a total of 32 interviews were conducted across Colombia. The inclusion criteria for study participants were certified healthcare providers who had worked in ETCRs, health consultants who had participated in healthcare development in ETCRs, and FARC health promoters. FARC health promoter was defined as a FARC ex-combatant who was part of the FARC guerrilla, and had received informal medical training as a combat nurse during the conflict. These participants worked as healthcare providers for fellow FARC combatants during the war, providing primary medical care including diagnoses and medication, and occasionally performing surgeries and other procedures. Following the peace agreement, these combat nurses were unable to work in a similar capacity since they lacked formal certification in the Colombia system, despite all their training and experience. As such, FARC health promoters shifted their role to volunteering with the community as unpaid healthcare liaisons, providing community health education and helping other FARC ex-combatants to maneuver the complexities of the Colombian health system. These FARC ex-combatants live in the ETCRs with their communities, and many are seeking educational opportunities to work as certified nursing assistants for their communities.

Participants were recruited through a convenience snowball method by a mixed gender research team with experience in emergency care delivery. Interviews were conducted in Bogota, Vista Hermosa, Medellin, Puerto Asis, and Rionegro, Colombia. Thirty in person interviews were conducted with the participant and one researcher in a private, quiet location. Two interviews were conducted over the phone according to participant preferences. Consent was obtained verbally before beginning each interview, and responses were recorded. All interviews were conducted in Spanish and recorded with the EasyVoiceRecorder app, while interviewers manually recorded observational notes on the interview script. Recordings were collected on password protected cell phones, uploaded to a secure DropBox folder, and immediately deleted from the cell phone. De-identified written data were stored in a secure location. During data collection, researchers met periodically to determine emerging interview themes, and collection was completed once the research team determined that data saturation had occurred.

### Data analysis

Study participants were assigned a specific code used throughout qualitative analysis to guarantee anonymity. All recorded interviews were transcribed by hand in Spanish into Microsoft Word and were stored securely on an encrypted laptop. Codebook development and direct content analysis of the transcripts was conducted according to the qualitative team-based coding approach described by MacQueen et al. [19]. Following immersion into the transcripts, a codebook was developed by the lead investigator by identifying and labeling key sections of text aligning with the study’s objectives. After initial approval by the analysis team, the draft codebook was verified. This process involved two co-investigators using a repetitive process of increasing inter-coder agreement by independently analyzing the same transcript, followed by collaborative discussion to refine the codebook. After two rounds of coding and adjustment, an inter-coder reliability of 0.78 using Cohen’s Kappa (κ = .78) was considered acceptable [20]. Once the codebook was verified, full transcripts were imported to NVivo12, and coding was conducted by two study investigators according to guidelines of the same protocol. Once coded, healthcare barriers were categorized according to Julio Frenk’s Domains of Healthcare Access, a comprehensive framework which defines various stages along the chain of healthcare seeking, including healthcare needs, desires, seeking, initiation, and continuation of attention [21]. Transcripts were then re-analyzed with an inductive approach to identify themes that did not fit within the Domains of Healthcare Access, which were labeled “other barriers” in the results. The number of participants who mentioned a certain healthcare barrier was calculated and reported as a frequency of the total study population. The identified healthcare access barriers were labeled as specific or not specific to the FARC population, and presented as such in the results narrative. This determination was made by evaluating if a barrier uniquely or disproportionately affected FARC patients, when compared with other vulnerable, rural dwelling populations in Colombia. Quotes reflecting the major healthcare barriers, with a special focus for the FARC-specific barriers, were translated into English and selected to include a range of participants. Quotes were labeled with the participant’s health professional role and ETCR where they had cared for FARC. ETCR reporting was alternated for participants with more than one quote and multiple ETCR representation. For especially sensitive quotes, ETCR locations were excluded to reduce risk of identification. To ensure data reliability, the following designs were implemented: i) source triangulation by interviewing certified healthcare providers and FARC health promoters, who themselves are ex-combatants, ii) a thematic codebook was developed and verified by two members of the research team using independent and cooperative techniques, iii) consensus on final results and theme categorizations was agreed upon by all analysis members of the research team. Member checking was not undertaken, to leave the data in its original form. This study design and analysis adhere to COREQ guidelines for qualitative research [22].

## Results

Thirty-two interviews were conducted among FARC health promoters, certified healthcare providers, and healthcare consultants who have worked in ETCRs. Interviews ranged from 17 to 88 min, with an average of 45.7 min. Four participants preferred to not be recorded during the interview, all of whom were FARC health promoters.

### Demographics

**Fig. 1:**
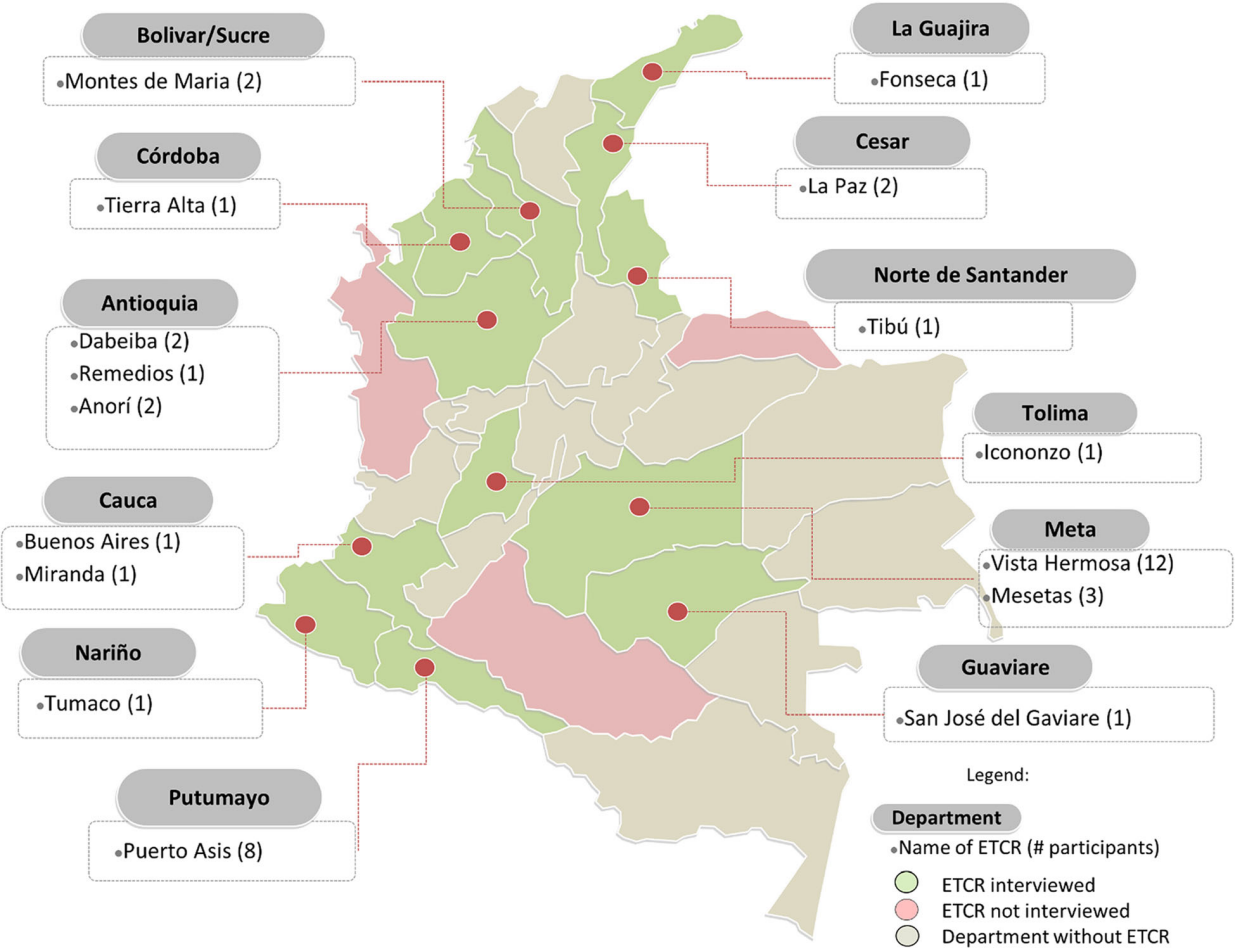
Espacios Territoriales de Capacitación y Reincorporación (ETCR) Locations of Healthcare Provider Experiences with FARC Ex-combatants. This figure demonstrates the 16 Espacios Territoriales de Capacitación y Reincorporación (ETCR), in 13 departments where our sample had worked with FARC ex-combatants. The ETCR names are according to the government designated name for the ETCRs, followed by the number of participants interviewed who had worked in that area in parenthesis. This figure was adapted from a template provided by yourfreetemplates.com, which granted permission for its modification and use

Participant demographics, including age, role and experiences with FARC communities are presented in Table 1. Participant ages ranged from 23 to 70 with an average of 36.4 years (SD = 11.5). The sample presented a variety of places of birth and residence, representing over 18 cities from 11 Departments. Two participants were born outside Colombia. Most worked in a clinical capacity, and one-third occupied additional roles as faculty, researchers or students (34%). Approximately one-fifth (21.9%) of the sample were FARC health promoters and had worked as combat nurses ranging from 2 to 15 years before the 2016 peace agreement. Our sample worked in 16 of the total 24 *Espacios Territoriales de Capacitación y Reincorporación* (ETCRs), spanning 13 departments across Colombia (Fig. 1).

**Table 1:** Participant Demographics

	Frequency (*n* = 32)	Percent
Gender		
Female	23	71.9%
Male	9	28.1%
Birthplace		
Urban	19	59.4%
Rural	13	40.6%
Residence		
Urban	18	56.3%
Rural	14	43.8%
Education level		
Primary	1	3.1%
Secondary	7	21.9%
Technical	6	18.8%
University	10	31.3%
Postgraduate (Specialization, Master’s, PhD)	7	21.9%
Role (2016–2019)		
FARC health promoter	7	21.9%
Physician	7	21.9%
Auxiliary nurse	6	18.8%
Nurse	5	15.6%
Health consultant	5	15.6%
Student	2	6.3%
Current role (multiple possible)		
Physician	8	25.0%
FARC health promoter	7	21.9%
Health consultant	7	21.9%
Auxiliary nurse	6	18.8%
Research role	6	18.8%
Nurse	4	12.5%
Physical therapist	1	3.1%
Has provided medical care for FARC ex-combatants	26	81.3%
ETCR	25	78.1%
Medical brigades	21	65.6%
Hospital	15	46.9%
Health center	12	37.5%
Worked with an interdisciplinary team in medical brigades	22	68.8%
Non-medical	12	37.5%
Lived with a FARC community	13	40.6%
Provided medical care to FARC combatants during the conflict	17	53.1%
Previous experience with humanitarian medicine	15	46.9%
Research among FARC communities	13	40.6%

This table presents demographics from our 32 participant sample, including age, gender, healthcare role, and experiences with FARC communities. *Abbreviations*: *FARC* Fuerzas Armadas Revolucionarias de Colombia, *ETCR* Espacio Territorial de Capacitación y Reincorporación

Both FARC health promoters and certified healthcare providers had experiences providing medical care to FARC ex-combatants (81.2%), ranging from a few days on short-term medical brigades, to living with the community for 2 years. Participants from both the FARC health promoter and certified healthcare professional samples had lived in ETCRs. While FARC health promoters lived with their communities since the implementation of the peace agreement 2 years prior, nurses often lived in ETCRs for periods of 3 months to work as the ETCR community health nurse. One FARC ex-combatant who worked as a surgeon during the conflict described the current role as a FARC health promoter, which began following the peace agreement:

“Right now, one is not able to do anything, not physically at least. I can help with orientation, guiding [FARC] in the city, saying ‘This is how you schedule a medical appointment; this is how the insurance works to receive authorizations.’ Orient them, bring them to the hospital, show them the system because they don’t know the city, the hospital management, or how to get an appointment.”

-FARC health promoter, Vista Hermosa, Meta.

Certified healthcare providers also reported experiences delivering medical care to FARC fighters who were part of the guerrilla during the conflict (53.1%). Nearly every healthcare professional who provided care to FARC combatants during the conflict was forced to do so. While some were kidnapped or had family killed by the guerrilla, many others were forced to deliver medical care under duress:

“It was horrible. One time a man showed up saying he needed a urinary catheter, but there weren’t any. He grabbed a revolver and fired bullets everywhere and looked through our supplies. He pointed the gun at my head and threatened me. It was horrible.”

– Nurse.

Brigades were funded by county health departments and local public hospitals (76.5%). Participants collaborated with many organizations, including the United Nations, local hospitals, universities, Colombian NGOs, and la *Agencia para la Reincorporación y Normalización* (ARN), the governmental organization responsible for overseeing FARC reincorporation.

### Barriers to healthcare access for FARC ex-combatants

**Fig. 2:**
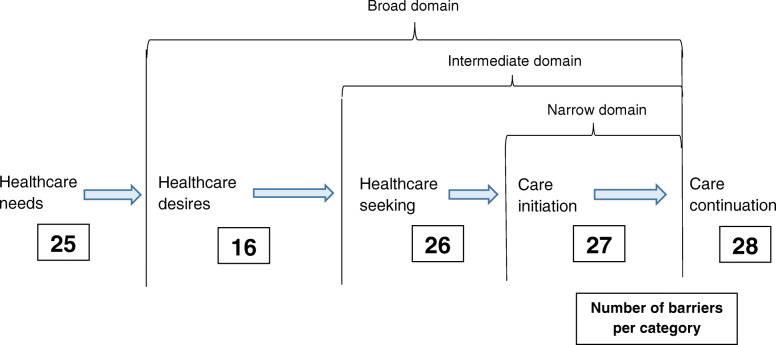
Healthcare Access Barriers for FARC Ex-combatants, according to Frenk’s Domains of Healthcare Access. Boxed numbers represent the number of barriers for each category of the total 141 identified barriers. Barriers which were classified as “other barriers,” including the social determinants of health and cultural factors are not included in this figure but are presented in Table 2 and Additional file 3

Across the diversity of ETCRs, participants identified a total of 141 barriers preventing FARC ex-combatants from receiving healthcare. These barriers are organized according to Frenk’s Domains of Healthcare Access (Fig. 2). Major categories are listed by frequency in Table 2 and expanded upon with quotes. Additional file 3 provides the listing and frequencies of all 141 barriers.

**Table 2:** FARC Healthcare Barrier Categories identified by ETCR healthcare providers and FARC health promoters

Barriers	n	%
**Healthcare Needs**		
Increased Incidence and complication of disease and trauma	31	96.9%
Increased Mental Health needs	28	87.5%
Pregnancy cases, maternal and prenatal care	16	50.0%
**Healthcare Desires**		
Lack of knowledge of the Colombian health system	30	93.8%
Transitioning from the FARC Health System	19	59.4%
Lack of preventive medicine and public health	13	40.6%
Preference for and ease of accessibility of traditional medicine	11	34.4%
Patient non-adherence, tendency to skip brigades, appointments, or follow-up	8	25.0%
Providers are unaccommodating, only motivated financially	2	6.3%
**Healthcare Seeking**		
Resource insufficiency in rural areas	31	96.9%
Burdensome geographic distances between communities and nearest hospital	27	84.4%
Economic barriers	21	65.6%
No internet nor WiFi, and limited cell phone connectivity	11	34.4%
Delayed or absent emergency health services	10	31.3%
Transport barriers	9	28.1%
**Healthcare Initiation**		
Stigma	29	90.6%
Health insurance (EPS) and healthcare initiation barriers	29	90.6%
FARC are slow to trust healthcare providers for fear of mistreatment	17	53.1%
Confusing bureaucratic processes, lacking information to understand how the healthcare system is constructed	15	46.9%
Identification associated problems in healthcare initiation	14	43.8%
**Healthcare Continuation**		
Medical brigades provided to ETCRs and surrounding rural communities	22	68.8%
FARC health promoters with valuable skills are not utilized as care providers	18	56.3%
Community providers, ETCR leaders lack communication channels to share common obstacles or create institutional change	12	37.5%
Wait-times on days of scheduled appointments	12	37.5%
Lack of clear avenues for FARC and surrounding communities to self-advocate	8	25.0%
EPS authorization delays cause some to not request them in the first place	7	21.9%
Loss to follow-up for lack of health system knowledge (labs, diagnostic exams)	7	21.9%
Migration of FARC community members and their families	6	18.8%
Inopportune appointment scheduling, compounded by care seeking barriers	4	12.5%
Non-adherence to medical treatment (taking medicines, vitamins, exercising)	2	6.3%
**Other Barriers**		
The social determinants of health	29	90.6%
Breach of the Peace Agreement by the government	18	56.3%
Education of forgiveness is required for reconciliation and ending cycles of violence	13	40.6%
Interventions lack holistic health approaches and psychosocial dimensions	6	18.8%
Research without interventions and uncoordinated care from universities and NGOs has created distrust of external groups among FARC	4	12.5%

This table summarizes the major barriers to FARC healthcare access, categorized according to Frenk’s Domains of Healthcare Access. A full list of barriers with subheadings and frequencies can be found in Additional file 3. *Abbreviations*: *ETCR* Espacio Territorial de Capacitación y Reincorporación, *ARN* Agencia para la Reincorporación y Normalización, *EPS* Entidad Promotora de Salud, *FARC* Fuerzas Armadas Revolucionarias de Colombia

#### Healthcare access barriers not specific to FARC ex-combatants

##### Transport barriers

Participants identified many healthcare access barriers that affected both FARC ex-combatants, and other vulnerable, rural-dwelling, and poor Colombian populations. Firstly, many ETCRs and rural communities face significant healthcare barriers due to geographic distance of care centers, lack of resources, and economic cost of transport. Twenty-seven participants stated that distance and natural geographic barriers created obstacles to reaching health centers. Most of the 24 ETCRs are geographically isolated and positioned anywhere from 1 to 8 h via ground transport from advanced care centers. FARC in the ETCR of Putumayo cross a river in canoe and travel over unpaved roads for an hour to reach the nearest hospital. Beyond that, it is common for rural hospitals to refer patients to tertiary centers in larger urban cities for specialty consultation, resulting in eight additional hours of travel on winding roads to arrive to the nearest advanced care center. This process often results in delayed care, as one FARC leader described the perception of accessing healthcare from a rural community:

“The process often feels like a game of ‘pass the dead,’ because no one wants or has the ability to take care of you. If you arrive to the hospital, you need to say that you are dying so they help you quickly. And if they don’t help you there, they send you to Pasto, because in our community there is no advanced hospital. They can send you to a good hospital, but even there the care is not very good.”

-FARC health promoter and ETCR leader.

FARC communities lacked safe and reliable transport options which delayed emergency care responses. Most ETCRs are in former or ongoing conflict areas with poor transport infrastructure, and FARC rely on non-medically trained motorcycle drivers. Poor road infrastructure led to increased road-traffic incidents and traumatic injuries. Transport barriers also affected medical providers’ ability to reach these communities for brigades and medical emergencies:

“Yes, I have to come daily to the ETCR. When it rains, I need to arrive by foot, and leave the ambulance in the mud.”

-Community health nurse, Vista Hermosa, Meta.

##### Financial barriers

Participants reported economic burdens associated with specialty vehicles traveling extensive distances. Lodging and food expenses in major urban centers presented an added economic burden. One physician in Antioquia described how a family could spend 240,000 pesos (80USD) in transport costs to a primary care center, about one-third of the minimum monthly salary.

##### Resource insufficiency barriers

Participants stated that healthcare resources in rural areas were insufficient to manage FARC and general population healthcare needs, including healthcare personnel, medications, and medical imaging technologies including X-ray machines and laboratories. Even when medications were available, incomplete diagnostic tools prevented treatment:

“The issue is complicated because even if I want to run certain tests, we can’t do any of them because we don’t have a laboratory. So what good will it do me to prescribe medicines without a lab or X-rays. We are missing a complete health system framework that allows for proper diagnoses and treatment.”

-Physician, Vista Hermosa, Meta.

ETCRs were supplied with an ambulance and small consultation room but lacked the personnel to staff these resources, including medical specialists to manage complex patients. In some ETCRs, an auxiliary nurse lived with the community for 3 months, but inconsistency of skilled providers resulted in preventable morbidity and mortality:

“It is very difficult for the people working in the ETCRs to make a diagnosis. Recently, a neonate died from sepsis. The baby had four days of fever, but when she was taken to the health center, the nurse’s diagnosis was to give the baby acetaminophen. If I was in her position, I would transport the baby to a hospital immediately. After significant delays, the community convinced the nurse to transport the baby, but she died on the way.”

-Physician.

Participants in Meta (*n* = 15) where most likely to mention resource insufficiency as the greatest problem affecting the FARC community. While this barrier was shared among nearly every participant in each region, Meta participants expanded upon it in greater detail than participants from other geographic areas.

##### Health system knowledge barriers

Health system knowledge deficiency was another barrier noted by participants. The majority of FARC were inexperienced with the processes to access care, including health insurance affiliations, acquiring medications, and referrals to specialists. When encountering barriers, FARC contact the *Agencia para la Reincorporación y Normalización* (ARN) health representative for their ETCR, a government employee responsible for assisting their healthcare process. However, there is no education for FARC patients, so they rarely learn how to overcome barriers for themselves. Almost all participants identified limited knowledge of the Colombian public health system as a barrier, not only for FARC but the population at large.

“I think that in Colombia not even the most educated people know how to use their health insurance. People misuse the system and access emergency services for things that aren’t urgent but die from things that are emergencies in low-resource areas because they lack access. It is difficult to say that FARC have less knowledge because almost nobody in Colombia knows the appropriate ways to access and use the health system.”

-Physician, Tierra Alta, Córdoba.

##### Health insurance barriers, general

Participants emphasized that access issues went beyond individual knowledge levels, pointing instead to flaws of the health system. They criticized the EPSs as a business model which favored lucrative and reactive care to preventive medicine, believing this EPS approach generated high costs for patients and the health system:

“I think there is an important task that the system has in this moment, and that is to strengthen the actions in Primary Health Care. That is key so that the community can end up accessing services. But this exercise has had difficulties in the rural areas, one of the reasons being because we have too many insurers, the EPS.”

- Physician and NGO director, Montes de María, Bolívar/Sucre.

Even when affiliated, authorization delays from EPS delayed care and required already overburdened physicians to appeal on behalf of their patients. The delays and lack of preventative care approaches from the EPS system were perceived as a major barrier to healthcare access by FARC health promoters and healthcare professionals. Finally, nearly every participant (90.6%) stated the importance of the social determinants of health. Key aspects included nutrition, water, and employment (Table 2), all of which our sample argued affect the health outcomes of FARC and other rural populations. One health consultant from Putumayo attributed elevated cases of gastroenteritis and malnutrition in the community to diminished focus on these factors. The lack of public health measures and infrastructure to support the social determinates of health led to increased rates of Leishmaniasis, pediatric diarrhea, and infectious diseases among these rural populations.

#### Healthcare access barriers specific to FARC ex-combatants

##### Special needs populations within FARC

Participants highlighted healthcare access barriers that specifically or disproportionately affected FARC ex-combatants. The sample perceived an increased incidence of unique needs for special populations including pregnant Farian women, FARC with physical disabilities, and FARC with mental health needs. They perceived a drastic increase in the number of Farian women becoming pregnant and having children following their demobilization. However, these providers cited multiple factors for high risk pregnancies, including history of multiple abortions, limited knowledge of prenatal care, and lack of access to obstetrical specialists:

“The health situation is very worrisome because we have many women here in the area, many pregnant girls with many babies. The pregnant women have to leave the space and spend two- or three-days seeking care because they are not attended to immediately.”

- Health consultant and social leader, Vista Hermosa, Meta.

Another specific population mentioned frequently were FARC with disabilities from trauma sustained during the conflict. Amputations, bone fractures, and other extremity deformities were common among combatants and citizens involved in fighting. However, while a citizen may have had access to medical care at the time of injury, FARC were mostly treated by fellow combatants in field hospitals. One physician described:

“Many have war injuries that need to be seen and evaluated by specialists, mainly orthopedics, dermatology, and general surgery. There are many orthopedic issues. There are some patients with amputations who have shells, bullets, or parts of mortar grenades still in their bodies. There are also complications because during the war, the FARC did their procedures in mobile hospitals. So these amputees are left with deformities because they weren’t amputated well.”

- Physician and hospital director, Vista Hermosa, Meta.

FARC with disabilities were reported to have a more difficult time securing the health insurance guaranteed to them by the peace agreement. Many participants, especially those empathetic to the FARC mission, criticized the EPS for prioritizing profit over equitable distribution of healthcare resources. The sample perceived a resistance on the part of the EPS to insure FARC with amputations or other disabilities, for the increased costs associated with caring for them:

“With disabilities there are many problems because the EPS did not want to cover ex-combatants with disabilities. So the EPS completely dissociated themselves from caring for this population and did not attend to them as a priority or with a differential approach.”

-Physical therapist and FARC member, Icononzo, Tolima.

Other diseases specific to the Farian community included musculoskeletal issues from years of joint stress, and the emergence of obesity, diabetes, hypertension, and malnutrition following the shift from active to sedentary lifestyle. A final special group of FARC highlighted by participants was those with psychological trauma and mental health needs. Participants perceived high needs for mental healthcare among FARC ex-combatants, who could benefit from medical management. Even those without diagnosable conditions underwent a drastic lifestyle changes in their reincorporation, which created a “psychosocial trauma,” generating a series of distrust, apprehension, and difficulties relating to others. The mental health needs of FARC ex-combatants ranged from this “psychosocial trauma” of reincorporation, to anxiety, depression, sleep disorders, and PTSD:

“I think there would be a high percentage of ex-combatants with psychological needs. When you change your life that drastically, it can cause a trauma. And to be involved in criminal activity for so long, that can cause a psychological trauma from the sense that you are always being persecuted. You don’t eat or sleep peacefully. You’re not sure if you’ll arrive home safely; it’s a trauma.”

- Community health nurse, Vista Hermosa, Meta.

Many reported that mental health services were under resourced and underutilized by FARC ex-combatants. One explanation for the discrepancy between needs and use was stigma of mental healthcare, as FARC believed they would be labeled as crazy. However, healthcare professionals involved in FARC community health outreach emphasized that this perception can change with proper education and approaching mental healthcare services as psychosocial attention framed as a process of accompaniment. The discrepancies between mental health needs and utilization were present in our own sample. The interviewed FARC health promoters were less likely to name mental health issues as a major problem among the FARC or report promoting these services to their communities when compared with the certified healthcare professionals.

##### Transition from the FARC healthcare system

Participants noted that healthcare reincorporation was complicated by transitioning from the FARC health system, which provided immediate medical attention without delays and did not require visiting a health center. During the conflict, FARC fighters had medical appointments scheduled for them by senior officers, including bi-annual physicals, vaccinations, and contraception administration [23]. Around half of FARC joined the guerrilla as minors, so most never learned the procedures to access the Colombian medical system:

“The FARC health system immediately resolved the medical difficulties that a patient had. They had a military combat logic, so medical care was always a rapid response. Now, FARC must transition from the logic of war to the logic of health insurance, which is administrative and bureaucratic. Many ex-combatants don’t know the healthcare access routes and are accustomed to a combat nurse resolving their health problems more simply than the health insurance companies are able to.”

-Physician and FARC member, Buenos Aires, Cauca.

Participants agreed that FARC members perceived state-based healthcare as less inviting and complain about provider hostility and wait-times. One FARC health promoter described a feeling of solidarity with FARC providers when being cared for in the FARC health system, since this person was part of the same organization. Conversely, this promoter described the Colombian health system as distressing to navigate, and lacking a trusting relationship with healthcare providers. FARC patients also had doubts surrounding their rights to access healthcare. Some did not know about the universal right to healthcare in Colombia, creating uncertainties as to whether they could access healthcare services. A physician from Antioquia stated that FARC patients were less likely to seek healthcare services and advocate for themselves while being cared for, since they had doubts regarding their rights for treatment.

##### Healthcare desires

Participants stated that FARC desires for healthcare services depend on their perspectives of wellness. Specifically, ex-combatants engaged in healthcare seeking only when they became sick, not for preventive measures. When FARC do seek care, healthcare professionals perceived them as likely to first try traditional medicine before accessing professional services. They believed this approach caused care delays and worsened these patients’ conditions. One nurse from Putumayo described how FARC ex-combatants do not prioritize their healthcare:

“The biggest problem is their own disinterest in healthcare. For having spent so many years in the countryside that they don’t want it themselves. They don’t come when we offer services, rather it is their custom to look for their own healers and self-medicate. They self-diagnose and seek treatment in their own culture.”

-Nurse and educator, Puerto Asis, Putumayo.

Healthcare professionals from Putumayo most often attributed FARC healthcare access barriers to a lack of personal desire. Many expressed that FARC were ungrateful for the healthcare offered to them, and only sought care for acute emergencies. Conversely, no FARC health promoters attributed culpability to an individual ex-combatant’s desire to seek wellness. These participants more often associated healthcare access difficulties to structural barriers, including financial, geographic, and administrative obstacles.

##### Discrimination barriers

Nearly all participants identified stigma as a barrier disproportionately affecting FARC ex-combatants. Stigma manifested from three sources: healthcare providers, communities surrounding ETCRs, and perceived stigma causing worry of poor treatment. Participants named stigma among healthcare professionals as an access barrier. Beyond explicit bias, healthcare professionals unconsciously viewed FARC as different from other patients. One physician from Vista Hermosa, Meta believed that while some feared FARC patients, others viewed them as a population to be treated differently. Though this physician empathized with those affected by the conflict, it also further excluded the FARC population. Stigma additionally existed from communities surrounding ETCRs, including a resentment towards special privileges received by FARC. One health consultant summarized:

“It is a matter of prejudice with the surrounding community. I think that there is a resistance in the communities surrounding the ETCRs to welcome ex-combatants. The people did not want to have the guerrillas close. We saw them like some monsters during the war, many of us still see them as monsters. As if they can’t be near us. From that community perspective, imagine what it will be like in the health system. There are health professionals who neither are ready to accept ex-combatants.”

-Health consultant, Fonseca, La Guajira.

Finally, participants reported that FARC were anxious they would be treated poorly in their healthcare interactions. A nurse from Puerto Asis, Putumayo expressed that FARC ex-combatants were very distrusting, and worried that healthcare personnel would harm them. As such, FARC preferred to remain anonymous in their healthcare process. Many refrained from disclosing personal information including true names and accurate past medical history to healthcare providers out of personal protection. While preserving this anonymity facilitated personal security, it created difficulties in establishing longitudinal care through accurate clinical histories, and building a trusted patient-provider relationship:

“There is no clinical history of past events, so there is no background of medical conditions, which can impede care. You need to start from zero with them because obviously they are not in the system.”

-Public health researcher, Dabeiba, Antioquia.

##### Identification barriers

Our sample reported that FARC patients experienced healthcare access difficulties due to identification issues. Most FARC left the guerrilla with no identification, which is required to access health insurance. Many FARC were given an alias when they entered the guerrilla, which they continued to use following demobilization. Issues of multiple names, no ID, and anonymity complicated health insurance registration:

“Identification is one of the biggest barriers. FARC had pseudonyms in the war, and the majority entered before turning 18 years old before getting an ID. There are many who still don’t have an ID and are afraid because they may appear as a political group and be exposed. Others have so many names that they go by.”

-Nurse, Anorí, Antioquia.

These identification barriers also affected children of FARC ex-combatants. One physician recounted the story of a FARC patient who had just birthed twins in a hospital, but only one child had health insurance affiliation due to the barrier of using multiple names.

“[She] had little twins just recently after the Peace Accord. One of them is affiliated and the other is not. Because one of them appears as her daughter with Mom’s given name, and the other appears as a son with her war name, which was not affiliated to health insurance. Despite them being twins! They were born on the same day in the same hospital, but their health insurance is different. The identification issue is super complicated for FARC.”

-Physician.

Healthcare professionals from Antioquia (*n* = 5) were the most likely to mention identification issues and the desire to remain anonymous as the most pressing healthcare access issue facing FARC ex-combatants. Participants from Meta, Putumayo, and Cauca rarely mentioned identification issues. When questioned, these participants believed most FARC ex-combatants in their regions had proper identification.

##### Health insurance barriers, FARC specific

Many FARC healthcare access issues stemmed from the vertical integration model of the EPS. For patients to receive non-urgent care, their EPS must be affiliated with the health facility where they are seeking care. However, many hospitals closest to the ETCRs were not affiliated with the insurer offered to FARC. This dilemma required FARC patients to unenroll from the government provided health insurance, and re-affiliate to another local provider to receive care. Participants across all geographic areas listed the vertical integration model of the EPS to affect FARC ex-combatants. Participants listed health insurance affiliation campaigns, expanding EPS coverage to the health centers closest to ETCRs, and the adoption of a partnership model between local and national EPSs as solutions to addressing insurance barriers. Migration is another issue complicating FARC healthcare access, especially with respect to EPS affiliation. Many FARC ex-combatants migrated from ETCRs in search of employment and better security, since many claim the government is not providing them basic services. However, migration created barriers in longitudinal care and EPS registration. EPSs are regionally affiliated, meaning that migratory FARC seeking healthcare in a department outside of their EPS affiliation could not access care. One FARC health promoter described this experience in the home region of Cúcuta:

“The hospital staff said to me: ‘We can’t help you. Your insurance affiliation is in Bogotá and you don’t have any affiliation in Cúcuta.’ So I think, ‘How could I be registered in Bogotá if I have lived my whole life in Cúcuta?’ Where you are affiliated becomes a barrier if you have moved, or the affiliation was done wrong, because there is no way to access health information or receive attention when it is needed.”

-FARC health promoter, Caño Indio, Norte de Santander.

##### Community engagement

One benefit to Farian communities were medical brigades by local hospitals. These brigades provided primary care to the ETCRs and surrounding areas and did not require health insurance to be evaluated by a physician. However, brigade frequencies varied without coordination between the hospitals and ETCRs. These changes resulted in ETCR health centers not always being staffed, and the FARC community being unaware of when medical staff would be present. Our sample reported low attendance and uncoordinated follow-up care between hospitals and ETCR leadership, which affected lab reporting and medication refills. Participants stated that hospitals have negative reputations in the ETCRs and emphasized the importance of working alongside FARC health promoters to coordinate community level care. Participants recommended contracting FARC health promoters in official roles through municipal health systems to strengthen ETCR and district hospital partnerships. Described by one physician:

“It is beautiful because there is a FARC health promoter who is one of the women that I admired most. She has very good empathy with the community. Things that people don’t tell the hospital staff, they tell her instead. She was trained in primary healthcare and is now working for the Hospital in Remedios. She makes medical consultation trips to the rural areas, because the hospitals have lost a lot of credibility in these areas, as you will realize happens in all the health centers in Colombia. The FARC don’t easily accept the people of the hospital because the lack of trust. But the FARC community accepts her, and she became the connection between the hospital and ETCR.”

-Physician, La Paz, Cesar.

Training and contracting FARC health promoters as primary healthcare workers was the most frequently mentioned potential solution to improve healthcare access. All interviewed FARC health promoters said they would like to participate in such a program.

##### Social determinants of health

Our sample believed that FARC healthcare access barriers were influenced by sociopolitical factors. FARC health promoters frequently blamed the Colombian government for healthcare access barriers facing FARC ex-combatants. Participants stated the government had not completed the healthcare promises in the peace agreement, including strengthening rural healthcare systems, and offering economic opportunities, security, and political participation:

“With the breach of the peace process, we see many difficulties, for example, with the viability of ‘productive projects.’ The persistence of paramilitaries and of armed dissidents generates distrust and creates an environment of persecution for the entire equality social movement that the FARC is promoting. The assassination of social leaders is very serious, and many ex-combatants have been murdered. This generates uncertainty and an absence of political freedom, which is what the FARC have always sought.”

-Physician and FARC member.

One physician who worked in Bolívar and Sucre explained that security issues limit positive healthcare seeking behavior. This participant stated that the increased murders of FARC ex-combatants caused many to feel insecure in the ETCRs. They perceived leaving the ETCR as a risk to their lives, including while seeking necessary medical care. This mentality presented problems when transferring patients for medical emergencies, resulting in delayed care. Finally, participants emphasized the need for a cultural change throughout Colombian society to encourage emotional healing. As Colombia has been affected by conflict for decades, participants emphasized that addressing structural health issues due to extended conflict required forgiveness. They recommended reconciliation as a component of peace building:

“We have a country that is very sick mentally. We don’t know how to live in community, because we can’t live with differences. We have all been touched by the violence, so we have a prejudice towards reconciliation. We didn’t have an appropriate pedagogy towards the peace agreements. We have a polarized country full of pain, which generates prejudices and barriers towards ex-combatants. We don’t see them like brothers, or people who can normalize and begin to heal.”

-Health consultant, Tumaco, Nariño.

## Discussion

This study provides a qualitative perspective of the barriers that reincorporating FARC ex-combatants face when accessing healthcare services. Studies show FARC ex-combatants living in ETCRs report less access to healthcare services, but little qualitative information was available to describe and categorize healthcare barriers. This study helps to fill this gap through in-depth interviews with ETCR community healthcare providers who are knowledgeable about the healthcare barriers of this population. For FARC ex-combatants, understanding health access barriers can help in designing targeted interventions and influencing policy aimed to address them. This information will be particularly useful, as FARC are reincorporating with high incidence of chronic and mental health conditions [11], and access to healthcare services was an integral part of the FARC Peace Agreement [8]. Additionally, this study adds to the growing literature on healthcare access barriers for reintegrating ex-combatant populations and provides useful findings in the Colombian context. Currently there are 17 ongoing reintegration processes run by the United Nations, where ex-combatants are beginning to access their country’s healthcare systems [24]. Findings on ex-combatant healthcare access often lead to policies which become implemented in future reintegration efforts and can be helpful in anticipating healthcare challenges for ex-combatant populations before they arise [25].

### FARC ex-combatant healthcare barriers

FARC ex-combatants living in ETCRs face a significant number of healthcare access barriers. Some barriers are similar to other rural-dwelling populations in Colombia, but others are specific to FARC ex-combatants. Participants emphasized geographic distances, non-adherence, low health system knowledge, lack of resources, and a failure to address the social determinants of health as obstacles to healthcare access among all rural populations living in low-resource areas, including ETCRs. Gaps in the health system infrastructure and educational system in rural areas have historically been ignored by the government [26]. However, there are certain barriers that disproportionately affect FARC ex-combatants. These include increased incidence and needs of special populations, stigma, transitions from the FARC health system, identification issues, and health insurance enrollment. Without addressing them, FARC ex-combatants could continue to struggle with these barriers, especially when the support of the ARN is withdrawn from the ETCRs. Data on FARC healthcare status supports this study’s claims for increased healthcare needs of FARC ex-combatants and special populations within this group, including pregnant Farian women, FARC with physical disabilities, and FARC with mental health needs. In the first FARC census, 3305 ex-combatants (33%) self-identified a chronic medical problem [4]. With prenatal care, approximately 2500 babies have been born to ex-combatants, with more than 600 living in ETCRs [27]. It is estimated that there are over 3000 FARC ex-combatants with physical disabilities, but there are delays to these patients receiving proper medical care [28]. Though FARC healthcare leaders have constructed a database with over 1200 FARC ex-combatants waiting for prosthetics in an attempt to increase their care access, academic reports continue to claim a lack of a differential approach to caring for FARC with disabilities from the Colombian health system [29]. Finally, preliminary reports have stated that there are FARC with significant psychological and mental health needs from the conflict [11]. Though widespread data on diagnosis frequency and burden of mental health conditions for FARC ex-combatants are not yet available, studies conducted with other demobilized armed groups in Colombia have demonstrated rates of PTSD above 50% [30]. Future studies could focus on comparing specific healthcare needs among these vulnerable subpopulations of FARC ex-combatants or differentiating healthcare access barriers and effective interventions for addressing them among these groups.

Our other findings on healthcare barriers are consistent with quantitative research conducted with FARC ex-combatants in ETCRs. Specifically, a quantitative survey administered to 591 FARC ex-combatants living in 23 ETCRs demonstrated a lack of prevention and promotion activities, as only 13.7% knew about the development of a community health monitoring system. While FARC patients were generally satisfied with emergency care, there was a lack of access to medications and lower knowledge about available health services. Less than two-thirds of respondents were able to access necessary medical care, including vaccinations (62.5%), ultrasounds (60.4%), and blood tests (50%). Finally, there was a need for increased health outreach, as only 17% of respondents had exposure to healthcare teams outside of the ETCR [16].

Using Julio Frenk’s Domains of Healthcare Access, the 141 identified barriers were categorized across the stages of healthcare access. This model allows for visualizing the burdens of these barriers along the healthcare process and determining approaches for interventions to address them. It showed that healthcare barriers affecting FARC ex-combatants are evenly distributed across the healthcare phases of needs, seeking, initiation, and continuation. Therefore, interventions must use a comprehensive approach to address the multiple needs described in each phase. Unilaterally addressing one barrier may result in continued inaccessibility for ex-combatants due to other barriers later in the process. The healthcare desires stage was a lower outlier, which included factors such as patients’ limited health knowledge, disinterest, or non-adherence. This finding is helpful for understanding that most barriers affecting FARC ex-combatant care access are related to system-level factors, rather than a lack of internal motivation.

Barriers in the different phases can compound one another, making access to care even more difficult. For example, delays due to identification barriers were perceived by FARC as stigma from providers. This made FARC less likely to resolve their identification and verification issues to access professional care in the future. Similarly, FARC ex-combatants who felt unsafe leaving ETCRs were less likely to seek services when geographic barriers to access were so great [31]. Finally, the economic cost of transport prevented pregnant Farian women from accessing prenatal care, leading to higher risk pregnancies. High risk pregnancies increased the need for emergency transport, which led to greater incurred costs. Using Frenk’s Domains of Healthcare Access framework allows for an individualized understanding of these barriers, and the need for a holistic approach to address them.

A surprising finding was barriers related to health insurance. According to ARN data from December 2019, 98% of FARC ex-combatants were affiliated to a health insurance provider [32]. This discrepancy could be explained by complexities of health insurance seen within our sample, including activating membership, multiple name registration, migration away from coverage locations, and uncontracted health centers. These problems could persist despite high rates of affiliation or member enrollment. A key issue in the EPS model is that most FARC members were affiliated to a national health insurance provider with contracts in urban centers far from ETCRs. With the EPS model of vertical integration [33], most FARC had to travel long distances to urban centers to receive care covered by their insurance. It is not clear why EPSs don’t expand coverage to the 24 hospitals closest to each of the ETCRs or establish partnerships with local EPSs to subsidize care for their affiliates. Another unexpected claim was stigma from healthcare providers. A pilot study evaluating Colombian emergency medicine physicians’ perceptions of FARC showed low explicit bias towards this population [34]. Despite this, the perception of stigma still exists as a care barrier. Healthcare worker-led community initiatives in ETCRs aimed at welcoming ex-combatants into healthcare could show that provider stigma should not be a concern for FARC.

A benefit to this in-depth qualitative research was identifying inconsistencies and discrepancies among our sample. We found that different healthcare access barriers were perceived as more pressing across varied geographic areas. Specifically, those in Meta emphasized resource and healthcare personnel insufficiency; participants in Antioquia highlighted identification issues and FARC desire for anonymity; and those in Putumayo placed culpability with individual FARC patients’ lack of desire for professional medical care as the most important barrier. These differences across geography are reasonable, since Colombia is such a diverse country with healthcare access issues that vary across geography, populations, and culture. Generally, healthcare professionals and FARC health promoters agreed on the healthcare access barriers but differed in their perception of how greatly each affected FARC ex-combatants. One noticeable discrepancy was the tendency to blame individual FARC ex-combatants for lack of seeking care, a perception mentioned frequently by healthcare professionals in Putumayo, but rarely stated by FARC health promoters. While FARC ex-combatants are often viewed as a homogenous population with universally shared healthcare issues, these results suggest there may be variations in healthcare needs and access barriers according to geography. Nearly all participants shared the belief that the government should be doing more for FARC ex-combatants to increase healthcare access, and that there has been a delay in implementation of promises outlined in the peace agreement. These findings present opportunities for future studies or interventions, including comparative studies focusing on distinguishing healthcare access barriers for FARC ex-combatants between various geographic areas, how provider perceptions of healthcare responsibility influence their perception of vulnerable patients, and differences in healthcare quality and outcomes for FARC patients before and after the peace agreement.

There were some expected barriers that were not mentioned by our sample. No participants expressed the cost of receiving healthcare services as burdensome. Colombia’s public health system provides 95% insurance coverage to its population [35] and ranks as one of the world’s most affordable for low-income citizens [36]. Instead, obstacles in accessing care contributed most strongly to barriers. No healthcare provider participants complained about their salary, but many expressed worries around personal safety working in conflict areas. This emphasis demonstrates that guaranteeing safe working conditions should be a priority for these care providers. Finally, participants’ responses elicited potential interventions to address healthcare access barriers among FARC ex-combatants. These include: (i) National EPSs partnering with the local EPSs close to ETCRs to allow FARC to access nearby hospitals; (ii) approach mental health services as psychosocial accompaniment to reduce stigma.; (iii) reinforce the social determinants of health including clean water, nutritious food, safety, and employment opportunities, and (iv) contract FARC health promoters as primary healthcare workers.

### Ex-combatant healthcare reintegration

The United Nations has emphasized the importance of guaranteeing access to healthcare services for reintegrating ex-combatants. Our study is important because there is a lack of research describing healthcare access barriers for reintegrating ex-combatants, and even less qualitative information. These findings add qualitative knowledge of healthcare barriers for ex-combatants, which can be helpful for designing healthcare programs for future reintegration processes. Increased mental health needs have been well-documented among many ex-combatant populations, including in Liberia [8], Rwanda [37], Somalia [38], and the Democratic Republic of the Congo (DRC) [39]. However, there often is a scarcity of mental health providers and services in post-conflict countries [3]. Our findings reflect this increased mental healthcare need and absence of adequate services and personnel. However, there are ongoing efforts with Colombian ex-combatants to better understand aggression, PTSD, and emotional processing [40], and recommendations have been made for mental health interventions [41]. Studies among ex-combatants in Bosnia, Haiti, Liberia and the DRC show that healthcare reintegration has failed to provide prevention and promotion activities or education for HIV and other infectious diseases [42]. This reflects our findings of limited health knowledge and lack of promotional health opportunities. Our findings show consistencies among healthcare barriers of other ex-combatant populations. In Uganda, ex-combatants were more likely to have chronic pain, and healthcare access was shown to be a factor in successful reintegration [43]. Research among adolescent ex-combatants in Sierra Leone demonstrated that resource insufficiencies and a lack of government support were the main factors attributed to healthcare access barriers [44]. Studies with Rwandan ex-combatants showed limited knowledge of healthcare systems and access [45]. In Liberia, healthcare access barriers for ex-combatants included distance, resource insufficiency and a lack of payment ability [46]. Increased needs, lack of resources, absence of government support, limited knowledge, and distance were common barriers shared in our findings. Lack of payment ability was not an issue reported by our sample. The universal health coverage of the Colombian health system is unique in its ability to provide coverage to ex-combatants compared with other settings, but problems persist in health insurance utilization. Compared with other ex-combatant populations, our sample differed in its mention of identification issues, transitions from conflict health systems, and insurance affiliation as healthcare access barriers. These barriers could merit further exploration in other reintegration processes. Finally, the potential solutions from our participants reflect interventions that have been successful in other settings. Reintegration programs in Cambodia, Indonesia, and Bosnia showed that building local capacity at ex-combatant reintegration sites increases access to healthcare services [47]. A similar effort could be explored through FARC health promoter training in ETCRs or expanding EPS coverage.

### Limitations

This study had several limitations. There was a small sample size but achieving data saturation and geographic diversity helped to have a characteristic population to draw our conclusions. Qualitative research lacks quantitative measures which are important for understanding an issue more comprehensively. This research can be a helpful framework for where to focus more specific, quantitative measures. Our sample had unequal representation between FARC health promoters and certified healthcare professionals, with only about one-fourth of participants identifying as members of the FARC. Similarly, our sample included FARC health promoters but did not interview FARC community members without experience providing healthcare services or education. This may have resulted in data gaps from a FARC community perspective. Future research could heighten the voices of the FARC health promoters, FARC leaders, and FARC ex-combatant community members. Finally, though a wide range of geographic areas were represented, participant experiences were not evenly distributed across them, and 8 ETCRs from 3 departments were not represented. Many of these areas have ongoing conflict and may face even more burdensome healthcare access barriers.

### Future directions

There are tangible interventions that could be implemented to increase healthcare access, related to health insurance usage, mental healthcare stigma, emergency services, and empowering FARC health promoters as certified primary healthcare providers. Training FARC health promoters as primary healthcare workers was the most frequently mentioned solution from participants. FARC health promoters defined healthcare barriers at equal rates to certified providers, demonstrating a comprehensive understanding of their community’s health challenges. FARC health promoters desired to serve their communities, and already possessed clinical skills beyond the scope of most community health workers [48]. Officially training these health promoters as primary healthcare providers for ETCRs may be a feasible intervention and could introduce opportunities for them to deliver healthcare beyond the scope of a typical community health worker. Contracting FARC health promoters through regional hospitals or EPSs could establish a health system which addresses many of the identified barriers. Specifically, this could (i) build trust between FARC and hospital personnel by placing trusted caregivers as first-line providers, (ii) formalize connections between ETCRs and hospitals, (iii) reduce care delays exacerbated by cultural misunderstandings, and (iv) strengthen a rural, emergency healthcare workforce by placing trained providers in proximity to ETCRs. The ARN has committed to post-conflict area development in ETCRs with the *Programas de Desarrollo con Enfoque Territorial* (PDET), with specific focuses on rural healthcare [49]. Community health workers have been shown to expand health coverage and promote peace in low-resource settings, including rural Colombia [50]. FARC health promoters are uniquely positioned with clinical skills, cultural competency, and desire to serve as community advocates to actualize the healthcare components of the *Programas de Desarrollo con Enfoque Territorial*. Other research could quantify many of the healthcare barriers outlined in this study, or the feasibility of EPS partnerships with local health centers or contracting FARC health promoters.

## Conclusion

This study provides a qualitative perspective of the barriers that reincorporating FARC ex-combatants face when accessing healthcare services. We found that FARC ex-combatants living in ETCRs face significant healthcare access barriers. These barriers included geographic distances, limited health system knowledge, a lack of resources, stigma, transitions from the FARC health system, identification issues, health insurance affiliations, and lack of the social determinants of health. Few studies have defined and categorized the specific barriers for reintegrating ex-combatants, and very little exists in the form of qualitative studies to describe them. This qualitative study used a novel approach, soliciting the perspectives of FARC health promoters and certified healthcare providers working in the ETCRs across Colombia. We used the Frenk’s Domains of Healthcare Access framework as a model to better understand the distribution of barriers across the different stages of the healthcare process and found that barriers were evenly distributed among each phase of healthcare access. This model could be a helpful framework to consider possible interventions. Addressing these gaps could help with successful FARC ex-combatant healthcare reincorporation and maintaining of the peace process in Colombia. Future studies are needed to quantify the healthcare barriers affecting FARC ex-combatants, differentiate access barriers among geographic areas and vulnerable subpopulations of FARC ex-combatants, and to pilot targeted interventions to improve healthcare access.

## Supplementary Information

**Additional file 1.** Interview script for healthcare providers of FARC ex-combatants. The qualitative script used for interviewing FARC ex-combatants and their healthcare providers.

**Additional file 2.** Interview for Healthcare Providers of Ex-combatants.

**Additional file 3.** FARC Healthcare Barriers identified by ETCR healthcare providers and FARC health promoters. The complete list of healthcare access barriers reported by participants with corresponding quantitative frequencies according to number of participants identifying each barrier.

## Data Availability

The datasets used and/or analyzed during the current study are available from the corresponding author on reasonable request.

## Abbreviations

FARC: Fuerzas Armadas Revolucionarias de Colombia
EPS: Entidad Promotora de Salud
ETCR: Espacio Territorial de Capacitación y Reincorporación
SRQR: Standard in Reporting in Qualitative Research
ARN: Agencia para la Reincorporación y Normalización
CNR: Consejo Nacional de la Reincorporación
DRC: Democratic Republic of the Congo
PDET: Programas de Desarrollo con Enfoque Territorial
