# Supplementary material for: Healthcare access barriers for FARC ex-combatants in Colombia: qualitative perspectives from healthcare providers and FARC health promoters
Source: BMC Public Health. 2021 Jan 8;21:102. doi: 10.1186/s12889-020-10062-3 (PMC7792039; doi:10.1186/s12889-020-10062-3)
Supplement: Supplementary file 1 — Additional file 1. Interview script for healthcare providers of FARC ex-combatants. The qualitative script used for interviewing FARC ex-combatants and their healthcare providers. [file 12889_2020_10062_MOESM1_ESM.pdf]

Entrevista para Proveedores de Salud de los Excombatientes

¿Ha leido el consentimiento informado?
- ◯ Si
- ◯ No

¿Tiene alguna pregunta en este momento?
- ◯ Si
- ◯ No

¿Le gustaría participar?
- ◯ Si
- ◯ No

SECTION 1:
Aspectos demográficos y validación

¿Cuántos años cumplidos tiene? ________________________________

¿Es Usted hombre o mujer? (mejor no preguntar, solo marcar)
- ◯ Hombre
- ◯ Mujer

¿Dónde nació? ________________________________

¿Dónde vive actualmente? ________________________________

¿Cuál o cuáles de las siguientes actividades ha desarrollado entre los años 2016 y 2018?
- ☐ Proveedor / promotor de salud de las FARC
- ☐ Medico/a (especialista, general, residente)
- ☐ Enfermero/a
- ☐ Odontólogo/a
- ☐ Docente / Facultad
- ☐ Investigador/a
- ☐ Otro

¿Cuál actividad desempeña actualmente?
- ☐ Proveedor / promotor de salud de las FARC
- ☐ Medico/a (especialista, general, residente)
- ☐ Enfermero/a
- ☐ Odontólogo/a
- ☐ Docente / Facultad
- ☐ Investigador/a
- ☐ Otro

¿Cuál es su nivel más alto de educación?
- ☐ Educación primaria
- ☐ Secundaria
- ☐ Técnica
- ☐ Profesional Universitario
- ☐ Maestría
- ☐ Doctorado
- ☐ Otro

¿Ha prestado servicios de atención en salud a excombatientes de las FARC?
- ◯ Si
- ◯ No
- ◯ No responde

¿Hace cuánto? (hace cuanto prestó el servicio o si actualmente está) ________________________________

¿Por cuánto tiempo prestó el servicio? ________________________________

¿En qué lugar o lugares les prestó sus servicios?

☐ Hospital
☐ Centro de salud
☐ ETCR (Espacio Territorial de Capacitación y Reincorporación)
☐ Una brigada
☐ Otro

Ha prestado servicios de salud en una comunidad de excombatientes o un ETCR?

○ Si
○ No

¿Cual es el nombre del ETCR? (o la comunidad de excombatientes) __________________________________

¿En cuál departamento se queda este ETCR? __________________________________

¿Aproximadamente cuántas personas vivían allí? (Cuantos excombatientes viven en la comunidad donde estaba) __________________________________

¿Me podría describir cómo llegó a la comunidad? (En una brigada o trabajó/vivió allí mucho tiempo) __________________________________

Si una brigada o viaje: ¿De dónde provienen los fondos y el apoyo logístico para la brigada? __________________________________

¿Usted trabajó con un equipo interdisciplinario?

○ Si
○ No
○ Otro

Si es interdisciplinario, ¿qué otros profesionales o personal estaba involucrado? (en la prestacion de servicios de salud)

____

Si no era interdisciplinario, ¿por qué no?

____

¿Prestó algún tipo de atención en salud a los combatientes de las FARC durante el conflicto?

○ Si
○ No
○ Otro

Si la respuesta es "si,"
¿Podría describir la experiencia en un breve resumen?

____

¿Había hecho brigadas o había participado en programas de medicina humanitaria o internacional antes de trabajar con excombatientes? (incluyendo con poblaciones vulnerables)

○ Si
○ No
○ Otro

SECTION 2:
Determinar desde las perspectivas médicas profesionales, las posibles barreras de servicios de salud que los excombatientes de las FARC enfrentan.

¿Cuáles son las principales barreras a sistemas de salud que usted percibe para la comunidad de excombatientes?

____

¿Cuál es el mayor problema que enfrentan estos excombatientes para tener una atención médica adecuada? __________________________________

¿Cuáles son las mayores dudas o quejas de los excombatientes para utilizar el sistema de salud? __________________________________

¿Los excombatientes que usted atendió sabían cómo usar el sistema de aseguramiento con la Nueva EPS?

- ◯ Si
- ◯ No
- ◯ Otro

¿Cómo considera que es el nivel de conocimiento del sistema de salud que tienen los excombatientes de las FARC? __________________________________

Fuera de temas de salud, ¿qué barreras percibe como las más importantes para la reincorporación exitosa para un excombatiente? __________________________________

SECTION 3:
Determinar a partir de opiniones médicas profesionales los problemas médicos que enfrentan las FARC.

¿Cuáles son las enfermedades o dificultades que ha visto en esta comunidad? __________________________________

¿Cuál cree que es el porcentaje de excombatientes que tienen problemas médicos crónicos? ___________________________

¿Cuál cree que es el porcentaje de excombatientes que tienen problemas psicológicos o de salud mental (TEPT, ansiedad, depresión)? ___________________________

¿Siente que la comunidad excombatiente aprecia su trabajo y ayuda?

- ◯ Si
- ◯ No
- ◯ Otro

En la atención o la consulta que Usted les brinda, ¿qué tan fácil o difícil es obtener información de salud de esta comunidad? (Es fácil hablar sobre temas de salud con estos pacientes?) ___________________________

SECTION 4:
Determinar el estado del ETCR y las experiencias brindando salud en esa área.

¿Puede describir la adecuación de infraestructura de salud donde brindó atención? __________________________________

¿Hay suficientes suministros, insumos o medicamentos?

- ◯ Si
- ◯ No
- ◯ No sabe
- ◯ Otro

Si no, ¿cuál cree que será la forma de obtenerlos? ___________________________

¿Hay suficiente personal de salud para atender a esta población?

- ◯ Si
- ◯ No
- ◯ No sabe
- ◯ Otro

Si no, ¿Qué tipo de personal hace falta? ___________________________

Si no: ¿Cuál sería un número apropiado? ___________________________

Si no: ¿Usted tiene ideas de como reclutar más proveedores de salud?

____

¿Se requiere adaptar estrategias específicas para atender excombatientes, diferentes a las que se requieren con otros pacientes?

◯ Si
◯ No
◯ Otro

Si la respuesta es si: ¿Cuáles estrategias?

____

¿Cuáles han sido las principales barreras para prestar atención en salud en este ETCR?

____

¿Ha podido superar algunas barreras para atender a los excombatientes en este ETCR?

◯ Si
◯ No
◯ Otro

¿Hay alguna manera de comunicarse con los proveedores de salud en otros ETCR?

◯ Si
◯ No
◯ Otro

¿Sería útil crear algún grupo con proveedores de salud de esta y otras áreas?

◯ Si
◯ No
◯ Otro

¿Sabe si algo así ya existe?

____

¿Crees que algunas guías de atención médica específicamente para excombatientes podrían beneficiar a los proveedores de salud que trabajan con ellos?

◯ Si
◯ No
◯ Otro

Si dicen "si", ¿Que deben incluir estas guías?

____

SECTION 5:
Determinar cómo trabajar con excombatientes puede cambiar las perspectivas de los proveedores de salud sobre las FARC y otras poblaciones vulnerables

¿Qué razones tuvo para tomar la decisión de trabajar con esta poblacion (o en este ETCR?)

____

¿Cuál fue el mayor "sacrificio" personal que debió hacer para trabajar aquí?

____

¿Cómo considera que es la percepción general que los profesionales de la salud tienen de los excombatientes?

____

Para los que tienen opiniones negativas, ¿Habría algo que hacer para cambiar esa percepción?

____

¿Qué opina de los excombatientes de las FARC?

____

¿Su trabajo con esta comunidad ha cambiado su opinión de esta población? ◯ Si ◯ No ◯ Otro

Si la respuesta es "si": Cómo la ha cambiado. __________________________________

¿Recomendaría a un compañero que trabaja en esta área o con una población de excombatientes? ◯ Si ◯ No ◯ Otro

¿Cuáles son las mayores barreras para que los médicos presten servicios en ETCR? __________________________________

¿Qué cree que podría hacerse para motivar a otros médicos a trabajar con excombatientes? __________________________________

¿Cree que esta experiencia afecta su disposición para trabajar con otras poblaciones vulnerables o marginadas en el futuro? (Está más o menos dispuesto a trabajar con poblaciones vulnerables o marginadas) ◯ Si ◯ No ◯ Otro

¿Ha cambiado su perspectiva sobre la mejor manera de brindar servicios de salud a poblaciones vulnerables? ◯ Si ◯ No ◯ Otro

¿Quisiera participar en una jornada sobre cómo prestar servicios de salud a los excombatientes? ◯ Si ◯ No ◯ Otro

Si no tiene grado universitario, ¿le gustaría participar en un programa formal para formarse como enfermero(a) o medico(a)? ◯ Si ◯ No ◯ Otro ◯ No aplica

Si ya es parte de alguno de estos programas, como se llama? __________________________

SECTION 6:
Brindar recomendaciones tangibles sobre cómo mejorar el acceso a la atención médica de los excombatientes.

¿Qué se puede hacer para ayudar a mejorar la asistencia médica de los excombatientes? __________________________________

¿Basados en su experiencia en la atención a excombatientes, que recomendaciones haría a la Agencia de Reincorporación y Normalización (ARN), a las ONGs y a las universidades que trabajan con excombatientes sobre temas de la salud? __________________________________

SECTION 7:
Evaluar la factibilidad y beneficio de la investigación y oportunidades de enseñanza entre las comunidades de excombatientes

¿Ha participado en algún proyecto de investigación con excombatientes? ◯ Si ◯ No ◯ Otro

Si: ¿Puede contarme sobre el proyecto? __________________________________

Si: ¿Qué tan dispuestos percibió a los excombatientes para participar en el proyecto? __________________________

Si: ¿Cuáles cree que son las principales barreras para que Usted realice una investigación con esta población? __________________________________

Si: ¿Está trabajando en colaboración con otras organizaciones? __________________________________

Oportunidades de Tutoría / enseñanza: ¿Ha sido docente? ◯ Si ◯ No ◯ Otro

Si: ¿La oportunidad de interactuar con comunidades de excombatientes podría beneficiar la enseñanza de estudiantes? ◯ Si ◯ No ◯ Otro

Si: ¿Me podría explicar cómo puede beneficiar? __________________________________

¿Cree que la comunidad de excombatientes se sentiría cómoda con la presencia de estudiantes en su territorio? ◯ Si ◯ No ◯ Otro

¿Cree que los excombatientes quisieran compartir sus experiencias o vivencias con estudiantes de diversos programas? ◯ Si ◯ No ◯ Otro

Muchas gracias por su tiempo y ayuda!
