# Supplementary material for: Healthcare access barriers for FARC ex-combatants in Colombia: qualitative perspectives from healthcare providers and FARC health promoters
Source: BMC Public Health. 2021 Jan 8;21:102. doi: 10.1186/s12889-020-10062-3 (PMC7792039; doi:10.1186/s12889-020-10062-3)
Supplement: Supplementary file 2 — Additional file 2. Interview for Healthcare Providers of Ex-combatants. [file 12889_2020_10062_MOESM2_ESM.pdf]

Appendix 2: Interview for Healthcare Providers of Ex-combatants

English translation

1. Have you read the informed consent?
2. Do you have a question in this moment?
3. Would you like to participate?

Section 1: Demographics and validation

1. How old are you?
2. What is your gender?
3. Where were you born?
4. Where do you live currently?
5. Which of the following roles have you filled between 2016 and 2018?
    a. FARC health promoter
    b. Physician
    c. Nurse
    d. Dentist
    e. Professor / academic faculty
    f. Researcher
    g. Other
6. Which of the following roles do you currently occupy?
    a. FARC health promoter
    b. Physician
    c. Nurse
    d. Dentist
    e. Professor / academic faculty
    f. Researcher
    g. Other
7. What is your highest level of education?
    a. Elementary
    b. Secondary
    c. Technical
    d. University
    e. Master's
    f. Doctorate
    g. Other
8. Have you worked in healthcare services delivery for FARC ex-combatants?
9. How long ago?
10. For how long did you work with the FARC?
11. Where did you work with the FARC?
    a. Hospital
    b. Health center
    c. ETCR (Territory Space for Training and Reincorporation)

    d. Brigade
    e. Other
12. Have you delivered healthcare to an ex-combatant community or in an ETCR?
13. What is the name of the ETCR?
14. What department is the ETCR located in?
15. Approximately how many people live there?
16. Could you describe how you arrived to the community? (was it a brigade o did you work or live there for extended periods of time?)
    a. If a brigade, where did the funds and logistically help for the brigade come from?
17. Have you worked in an interdisciplinary team?
    a. If it is interdisciplinary, what other healthcare professionals were involved?
    b. If it was not interdisciplinary, why not?
18. Did you deliver healthcare to FARC combatants during the conflict?
    a. Could you describe the experience in a brief summary?
19. Had you participated in medical brigades or humanitarian medicine before working with ex-combatants? (including other vulnerable populations)

Section 2: Determine from the perspective of medical professionals the possible healthcare service barriers that face FARC ex-combatants

1. What are the main barriers to health systems that you perceive for the ex-combatant community?
2. What is the main problem that affect ex-combatants to have adequate medical attention?
3. What are the main doubts or complains that ex-combatants have to use the health system?
4. Did the ex-combatants that you worked with know how to use the Nueva EPS health insurance?
5. How is the knowledge level of FARC ex-combatants with regards to the house system?
6. Outside of healthcare, what barriers do you perceive as the most important for the successful reincorporation of an ex-combatant?

Section 3: Determine from the opinions of medical professionals the medical challenges that face FARC ex-combatants

1. What are the illnesses or difficulties that you have seen in the community?
2. What do you think is the percentage of ex-combatants with chronic medical problems?
3. What do you think is the percentage of ex-combatants with psychological or mental health needs (PTSD, anxiety, depression)?
4. Do you feel that the ex-combatant community appreciates your work and help?
5. In the attention you give, how easy or difficult is it to obtain health information from this community? (is it easy to speak about healthcare themes with these patients?)

Section 4: To determine the state of the ETCR and the experiences delivering healthcare in these areas

1. Could you describe the health infrastructure where you delivered healthcare?
2. Are there sufficient supplies, equipment, and medications?

    a. If no, what would be the way to obtain them?
3. Are there sufficient people to attend to the population?
    a. If no, what type of personnel are missing?
    b. If no, what would be the appropriate number?
    c. If no, do you have ideas of how to recruit more healthcare providers?
4. Does it require adapting specific strategies to work with ex-combatants, different from those with other patients?
    a. If yes, what strategies?
5. What have been the main barriers to deliver health attention in the ETCR?
6. Have you been able to overcome these barriers to care for the ex-combatants in the ETCR?
7. Is there a way to communicate with providers from other ETCRs?
8. Would it be useful to create a group with providers from this area and others?
9. Does something like this already exist?
10. Do you think some healthcare attention guides specifically for ex-combatants could benefit the health providers that work with them?
    a. If yes, what should these guides include?

Section 5: Determine how to work with ex-combatants can change the perspectives of healthcare providers about FARC and other vulnerable populations

1. What reasons did you decide to work with this population?
2. What was the biggest personal sacrifice that you made to work here?
3. How do you consider the general perception of healthcare providers towards ex-combatants?
    a. For those with negative opinions, is there a way to change this perception?
4. What do you think of FARC ex-combatants?
5. Has your work with this population changed your opinion of them?
6. Would you recommend to a colleague that they work with ex-combatants?
7. What are the major barriers to doctors delivering healthcare in ETCRs?
8. What do you believe can be done to motivate other doctors to work with ex-combatants?
9. Did this experience affect your willingness to work with other vulnerable populations in the future? (Are you more or less willing to work with marginalized or vulnerable groups?)
10. Has this experience changed your perspective about the best way to deliver healthcare services to vulnerable populations?
11. Would you like to participate in a workshop about how to deliver healthcare to ex-combatants?
12. If you do not have a professional degree, would you like to participate in a formal program to train as a nurse or doctor?
13. If you are already part of such a program, what is the name?

Section 6: Tangible recommendations about how to improve healthcare access of ex-combatants

1. What can be done to improve the healthcare attention of ex-combatants

2. Based on your experience in attending to ex-combatants, what recommendations what you give to the Agency for Reincorporation and Normalization, to NGOs, and universities that work with ex-combatants about how to improve their health?

Section 7: Evaluate the feasibility and benefit of research and teaching opportunities among ex-combatant communities

1. Have you participated in a research project with ex-combatants?
    a. If yes, can you tell me about the project?
    b. If yes, how willing were ex-combatants to participate in the project?
    c. If yes, what were the main barriers to being able to complete your project with this population?
    d. If yes, were you working in collaboration with other organizations?
2. Have you worked as a teacher, professor, or faculty member?
    a. If yes, could the opportunity to interact with ex-combatant communities benefit the learning of students?
    b. Could you explain to me how it would benefit?
    c. Would ex-combatant communities feel comfortable with the presence of students in their area?
    d. Would ex-combatants want to share their experiences or living arrangements with students from diverse programs?

Thank you for your time and help!
