# Supplementary material for: Healthcare access barriers for FARC ex-combatants in Colombia: qualitative perspectives from healthcare providers and FARC health promoters
Source: BMC Public Health. 2021 Jan 8;21:102. doi: 10.1186/s12889-020-10062-3 (PMC7792039; doi:10.1186/s12889-020-10062-3)
Supplement: Supplementary file 3 — Additional file 3. FARC Healthcare Barriers identified by ETCR healthcare providers and FARC health promoters. The complete list of healthcare access barriers reported by participants with corresponding quantitative frequencies according to number of participants identifying each barrier. [file 12889_2020_10062_MOESM3_ESM.docx]

| Appendix 3: Complete list of FARC Healthcare Barriers identified by ETCR healthcare providers and FARC health promoters | | |
| --- | --- | --- |
| Barriers | n | % |
| **Healthcare Needs** | | |
| Pregnancy cases, maternal and prenatal care | 16 | 50.0% |
| High risk pregnancies (no prenatal care, prior abortions) | 8 | 25.0% |
| Increased Incidence and complication of disease and trauma | 31 | 96.9% |
| Infectious and tropical diseases (dengue, malaria, Zika) | 16 | 50.0% |
| Complicated wartime amputations and injuries with improper management | 13 | 40.6% |
| Musculoskeletal complications | 10 | 31.3% |
| Dermatological conditions (Leishmaniosis, scarring) | 10 | 31.3% |
| Water-borne diseases, intestinal parasites, diarrhea, stomach pain | 10 | 31.3% |
| Chronic conditions (hypertension, diabetes) | 10 | 31.3% |
| Reproductive healthcare access, gynecology | 9 | 28.1% |
| Respiratory infections, difficulties | 7 | 21.9% |
| Malnutrition | 6 | 18.8% |
| Weight gain, obesity due to lifestyle changes | 4 | 12.5% |
| Ophthalmological conditions | 3 | 9.4% |
| Increased road traffic incidents due to lack of safe transportation | 3 | 9.4% |
| Sexually transmitted diseases, UTIs | 2 | 6.3% |
| Physical rehabilitation, physical therapy | 2 | 6.3% |
| Alcoholism | 1 | 3.1% |
| Increased Mental Health needs | 28 | 87.5% |
| Depression, anxiety, sleep problems, psychological effect, suicide | 20 | 62.5% |
| Resistance to seek mental healthcare services for associations with craziness | 13 | 40.6% |
| Psychosocial trauma from reincorporating to a new style of life | 10 | 31.3% |
| Stigma surrounding mental health services | 8 | 25.0% |
| Increased incidence of PTSD | 7 | 21.9% |
| Emotional and behavioral difficulties around reincorporation | 6 | 18.8% |
| **Healthcare Desires** | | |
| Lack of knowledge of the Colombian health system | 30 | 93.8% |
| Lack of knowledge to utilize EPS services | 27 | 84.4% |
| Lack of formal or effective training on accessing the health system | 14 | 43.8% |
| Dependence on the ARN for appointment scheduling and follow-up care | 13 | 40.6% |
| Not knowing one’s rights to be attended to in appropriate health centers | 5 | 15.6% |
| Lack of general health literacy | 8 | 25.0% |
| Transitioning from the FARC Health System | 19 | 59.4% |
| Need to actively seek care, no system of personal delivery | 12 | 37.5% |
| Necessary to personally schedule appointments | 10 | 31.3% |
| Misunderstanding/impatience of wait-times for authorizations, procedures | 18 | 56.3% |
| Impersonal services from medical professionals | 4 | 12.5% |
| Providers are unaccommodating, only motivated by money | 2 | 6.3% |
| Lack of preventative medicine and public health | 13 | 40.6% |
| Preference for and ease of accessibility of traditional medicine | 11 | 34.4% |
| Tendency to skip brigades, appointments, or testing follow-up | 8 | 25.0% |
| Not capitalizing on beneficial community healthcare programming | 5 | 15.6% |
| **Healthcare Seeking** | | |
| Resource insufficiency in rural areas | 31 | 96.9% |
| Medical personnel (medical, mental health, and public health specialists) | 30 | 93.8% |
| Delays in receiving specialized care due to shortage of specialists | 19 | 59.4% |
| Referral to tertiary health center due to lack of resources | 21 | 65.6% |
| Worsening conditions due to treatment delays during referral | 9 | 28.1% |
| Non-centralized medical records | 1 | 3.1% |
| Infrastructure (appropriate healthcare center, emergency medical services) | 20 | 62.5% |
| Medication | 19 | 59.4% |
| Technologies (labs, x-rays, medical imaging, refrigeration) | 12 | 37.5% |
| Burdensome geographic distances between communities and nearest hospital | 27 | 84.4% |
| Lack of infrastructure creating difficult travel conditions | 12 | 37.5% |
| Economic barriers | 21 | 65.6% |
| Co-pay, other expenditures not covered by EPS (psychologist) | 14 | 43.8% |
| Economic cost of transport to primary health center | 9 | 28.1% |
| In case of referral to tertiary healthcare center: | 8 | 25.0% |
| Cost of staying in major urban centers for complex care | 5 | 15.6% |
| Economic cost of transport | 4 | 12.5% |
| Time taken from employment and childcare to seek medical care | 4 | 12.5% |
| Transport barriers | 9 | 28.1% |
| Lack of personal vehicles and reliable transport options | 4 | 12.5% |
| Common to arrive to health centers on foot | 2 | 6.3% |
| Increased difficulty for those with mobility problems | 2 | 6.3% |
| No internet nor WiFi, and limited cell phone connectivity | 11 | 34.4% |
| Clinic appointments must be made in person | 5 | 15.6% |
| Internet is not reliable for appointment scheduling or determining care options | 1 | 3.1% |
| Delayed or absent emergency services | 10 | 31.3% |
| **Healthcare Initiation** | | |
| Health insurance (EPS) and healthcare initiation barriers | 29 | 90.6% |
| Delays in authorization from EPS national office (8 days-2 months) | 15 | 46.9% |
| Delays for appeals when EPS does not cover procedure, medication | 13 | 40.6% |
| FARC who are still unaffiliated to an EPS | 12 | 37.5% |
| FARC who are improperly registered under an alias or incorrect name | 9 | 28.1% |
| Inactivated EPS membership, despite affiliation | 6 | 18.8% |
| EPS not contracted with health center nearest to ETCR | 9 | 28.1% |
| Lack of knowledge to switch to more appropriate or accessible EPS | 9 | 28.1% |
| EPS refusal to cover elderly, pre-existing conditions, disabilities | 5 | 15.6% |
| Cannot access a specialist, prescribed medications without an EPS | 8 | 25.0% |
| Confusing bureaucratic processes, lacking information to understand how the healthcare system is constructed | 15 | 46.9% |
| Identification associated problems in healthcare initiation | 14 | 43.8% |
| Desires for some ex-combatants to remain anonymous, use an alias | 9 | 28.1% |
| Not owning official or state-recognized identification | 7 | 21.9% |
| Document falsification or failure to legalize | 5 | 15.6% |
| No medical history | 3 | 9.4% |
| Stigma | 29 | 90.6% |
| Stigma of healthcare providers towards FARC ex-combatants | 27 | 84.4% |
| Fear from healthcare providers forced by employer to work with FARC | 15 | 46.9% |
| Resentment towards FARC receive special privileges | 8 | 25.0% |
| Stigma of communities surrounding ETCRs towards FARC ex-combatants | 10 | 31.3% |
| Fear/stigma from FARC towards healthcare providers, community | 7 | 21.9% |
| Self-stigmatization of FARC communities (not deserving of care) | 1 | 3.1% |
| FARC are slow to trust healthcare providers for fear of how they may be treated | 17 | 53.1% |
| FARC are guarded about healthcare topics in consultations | 15 | 46.9% |
| Anxiety regarding past medical history (injuries, abortions, mental care) | 3 | 9.4% |
| Healthcare providers lack humanization and FARC cultural competency | 9 | 28.1% |
| **Healthcare Continuation** | | |
| Medical brigades provided to ETCRs and surrounding rural communities | 22 | 68.8% |
| Brigades are infrequent and frequency has diminished with time | 9 | 28.1% |
| Lack of coordination between hospital and ETCR leadership | 9 | 28.1% |
| Community members are unsure when brigades will come | 7 | 21.9% |
| Issues with longitudinal care, filling prescriptions, obtaining test results | 4 | 12.5% |
| Little to no focus on mental health, psychosocial reincorporation | 7 | 21.9% |
| FARC health promoters with valuable skills are not utilized as care providers | 18 | 56.3% |
| Community providers, ETCR leaders lack communication channels to share common obstacles or create institutional or macroscale change | 12 | 37.5% |
| Lack direct communication with ARN, CNR, regional health meetings | 9 | 28.1% |
| Cannot communicate common needs, healthcare programming ideas to EPS | 4 | 12.5% |
| Lack of clear avenues for FARC and surrounding communities to self-advocate | 8 | 25.0% |
| Physicians need to negotiate with EPS | 2 | 6.3% |
| No communication with NGOs, politicians, or international community | 7 | 21.9% |
| Wait-times on days of scheduled appointments | 12 | 37.5% |
| Lack of immediacy of services causes some to be lost to follow-up | 5 | 15.6% |
| EPS authorization delays cause some to not request them in the first place | 7 | 21.9% |
| Loss to follow-up for lack of health system knowledge (labs, diagnostic exams) | 7 | 21.9% |
| Inopportune appointment scheduling, compounded by care seeking barriers | 4 | 12.5% |
| Non-adherence to medical treatment (taking medicines, vitamins, exercising) | 2 | 6.3% |
| Migration of FARC community members and their families | 6 | 18.8% |
| Disease transmission between ETCR and local communities | 2 | 6.3% |
| Migration complicates brigade and local health center capacity operations | 1 | 3.1% |
| Barriers in receiving test results if they are not present the day of the brigade | 2 | 6.3% |
| Finding an EPS contracted health center, or affiliating to a new EPS | 5 | 15.6% |
| Incompletion of the Peace Agreement by the government | 18 | 56.3% |
| ETCRs continue to lack basic necessities for public health capacity building | 9 | 28.1% |
| No long-term healthcare capacitation plan | 6 | 18.8% |
| Gaps in longitudinal care as humanitarian aid organizations withdraw | 1 | 3.1% |
| **Other Barriers** | | |
| Social determinants of health | 29 | 90.6% |
| Education | 16 | 50.0% |
| Employment | 16 | 50.0% |
| Potable Water Access | 9 | 28.1% |
| Personal security, fear of harm, receiving threats | 6 | 18.8% |
| Land access | 6 | 18.8% |
| Income opportunities, managing finances | 5 | 15.6% |
| Community support | 4 | 12.5% |
| Access to nutritious food | 4 | 12.5% |
| Environmental pollution, disasters | 2 | 6.3% |
| Cultural reincorporation | 15 | 46.9% |
| Focus on, opportunities for psychosocial integration | 11 | 34.4% |
| ETCRs and preferential treatment further ostracize FARC communities | 2 | 6.3% |
| Some FARC ex-combatants prefer to remain outside of society | 8 | 25.0% |
| *Pedagogy of Peace* is required for reconciliation and ending cycles of violence | 13 | 40.6% |
| Self-perception as victims and aggressors perpetuates negative self-image | 8 | 25.0% |
| Not all Colombians are ready to forgive or move on from the conflict | 6 | 18.8% |
| Interventions lack holistic health approaches and psychosocial dimensions | 6 | 18.8% |
| Research without interventions and uncoordinated care from universities and NGOs has created distrust of external groups among FARC | 4 | 12.5% |
| This table demonstrates the total 141 barriers identified by participants to affect FARC healthcare access, and categorized according to Frenk’s Domains of Healthcare Access. Abbreviations: ETCR=Espacio Territorial de Capacitación y Reincorporación, ARN=Agencia para la Reincorporación y Normalización, EPS=Entidad Promotora de Salud, FARC=Fuerzas Armadas Revolucionarias de Colombia, FHP = FARC Health Promoters, CNR = Consejo Nacional de la Reincorporación | | |
